# Supplementary material for: Genomic Constellation of Human Rotavirus G8 Strains in Brazil over a 13-Year Period: Detection of the Novel Bovine-like G8P[8] Strains with the DS-1-like Backbone
Source: Viruses. 2023 Mar 1;15(3):664. doi: 10.3390/v15030664 (PMC10056101; doi:10.3390/v15030664)
Supplement: Supplementary file 1 [file viruses-15-00664-s001.zip › viruses-1984756-supplementary.pdf]

Supplement S1. Length and nucleotide position of each gene segment of the human rotavirus G8 strains detected in Brazil, 2007-2020.

				IAL-R193 G8P[8]		IAL-R558 G8P[8]		IAL-R2601 G8P[4]		IAL-R2600 G8P[4]		IAL-R2598 G8P[4]		IAL-R2597 G8P[4]		IAL-R2437 G8P[6]		IAL-R2404 G8P[6]		IAL-RN377 G8P[6]		IAL-RN374 G8P[6]		IAL-RN373 G8P[6]		IAL-RN361 G8P[6]	
**Gene segment**	**Size (nt)**	**Encoded protein**	**ORF position (nt)**	**Size (nt)**	**Position (nt)**	**Size (nt)**	**Position (nt)**	**Size (nt)**	**Position (nt)**	**Size (nt)**	**Position (nt)**	**Size (nt)**	**Position (nt)**	**Size (nt)**	**Position (nt)**	**Size (nt)**	**Position (nt)**	**Size (nt)**	**Position (nt)**	**Size (nt)**	**Position (nt)**	**Size (nt)**	**Position (nt)**	**Size (nt)**	**Position (nt)**	**Size (nt)**	**Position (nt)**
1	3,302	VP1	19-3,285	677	1-676	648	1-645	663	16-678	606	16-621	598	16-613	620	16-635	604	8-611	606	8-613	606	8-613	605	8-612	671	8-678	606	8-613
2	2,690	VP2	17-2,656	648	2-649	653	1-653	610	51-660	608	53-660	595	66-660	600	61-660	649	12-660	649	12-660	649	12-660	648	13-660	648	13-660	648	13-660
3	2,591	VP3	50-2,557	697	6-702	652	2-653	629	50-678	629	50-678	612	50-661	629	50-678	643	45-687	609	45-653	639	45-683	634	45-678	634	45-678	524	456-979
4	2,362	VP4	10-2,337	771	64-834	767	73-839	756	73-828	781	56-836	747	77-823	750	75-824	753	78-830	763	73-835	766	77-842	731	104-834	766	78-843	759	71-829
5	1,611	NSP1	11-1,492	770	612-1381	1451	62-1512	1512	10-1521	956	573-1528	928	582-1509	811	586-1396	947	575-1521	797	576-1372	994	573-1566	794	576-1369	770	572-1341	881	636-1516
6	1,356	VP6	24-1,217	1270	15-1284	1306	35-1340	1250	35-1284	1292	35-1326	1203	90-1292	1241	60-1300	1297	35-1328	1281	42-1322	1290	38-1327	1294	35-1328	1285	36-1320	1094	119-1212
7	1,104	NSP3	16-948	472	144-615	886	125-110	804	202-1005	765	221-985	804	202-1005	962	43-1004	938	40-977	918	60-977	960	47-1006	966	43-1008	967	40-1006	914	68-979
8	1,059	NSP2	30-983	429	257-685	683	328-1010	779	83-861	940	57-996	782	66-847	954	62-1015	923	79-1001	916	83-998	837	53-889	588	415-1002	930	61-988	863	124-986
9	1,062	VP7	49-1,029	914	75-988	711	1-711	879	55-933	449	106-554	930	65-994	925	29-953	925	29-953	801	194-994	944	58-1001	773	226-998	937	58-994	725	279-1003
10	751	NSP4	42-569	663	49-711	666	47-712	613	59-671	664	45-708	663	46-708	661	42-702	706	2-707	666	46-711	630	47-676	602	45-646	637	38-674	582	59-640
11	667	NSP5/6	5-598	612	1-612	667	1-667	667	1-667	667	1-667	667	1-667	667	1-667	667	1-667	667	1-667	667	1-667	667	1-667	667	1-667	667	1-667
**Total genome**	**18,474**	-	-	**7,923**	-	**9,090**	-	**9,192**	-	**8,357**	-	**8,529**	-	**8,820**	-	**9,052**	-	**8,673**	-	**8,982**	-	**8,302**	-	**8,912**	-	**8,263**	-

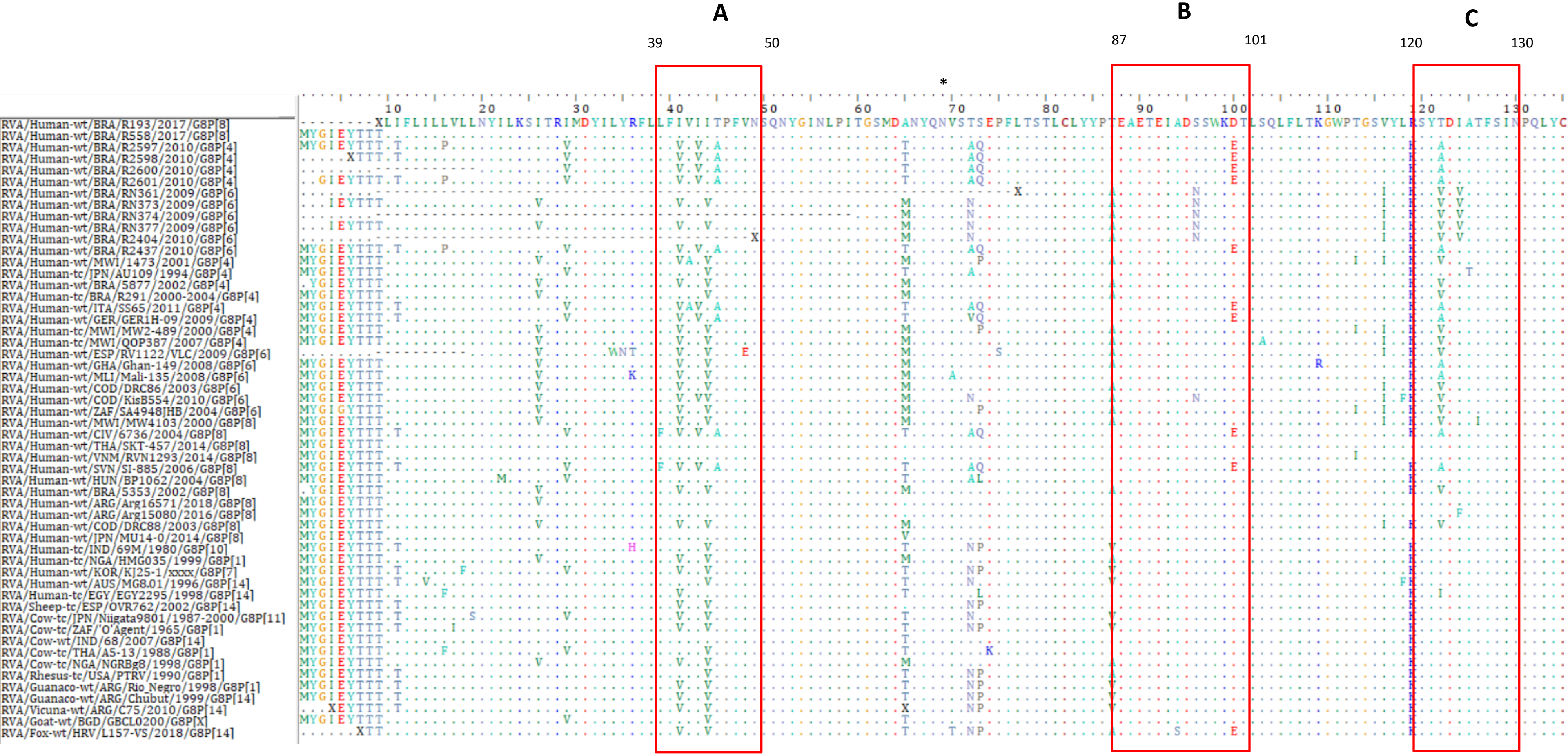
A
B
C
39
50
87
101
120
130
RVA/Human-wt/BRA/R193/2017/G8P[8]
RVA/Human-wt/BRA/R558/2017/G8P[8]
RVA/Human-wt/BRA/R2597/2010/G8P[4]
RVA/Human-wt/BRA/R2598/2010/G8P[4]
RVA/Human-wt/BRA/R2600/2010/G8P[4]
RVA/Human-wt/BRA/R2601/2010/G8P[4]
RVA/Human-wt/BRA/RN361/2009/G8P[6]
RVA/Human-wt/BRA/RN373/2009/G8P[6]
RVA/Human-wt/BRA/RN374/2009/G8P[6]
RVA/Human-wt/BRA/RN377/2009/G8P[6]
RVA/Human-wt/BRA/R2404/2010/G8P[6]
RVA/Human-wt/BRA/R2437/2010/G8P[6]
RVA/Human-wt/MWI/1473/2001/G8P[4]
RVA/Human-tc/JPN/AU109/1994/G8P[4]
RVA/Human-wt/BRA/5877/2002/G8P[4]
RVA/Human-tc/BRA/R291/2000-2004/G8P[4]
RVA/Human-wt/ITA/SS65/2011/G8P[4]
RVA/Human-wt/GER/GER1H-09/2009/G8P[4]
RVA/Human-tc/MWI/MW2-489/2000/G8P[4]
RVA/Human-tc/MWI/QOP387/2007/G8P[4]
RVA/Human-wt/ESP/RV1122/VLC/2009/G8P[6]
RVA/Human-wt/GHA/Ghan-149/2008/G8P[6]
RVA/Human-wt/MLI/Mali-135/2008/G8P[6]
RVA/Human-wt/COD/DRC86/2003/G8P[6]
RVA/Human-wt/COD/KisB554/2010/G8P[6]
RVA/Human-wt/ZAF/SA4948JHB/2004/G8P[6]
RVA/Human-wt/MWI/MW4103/2000/G8P[8]
RVA/Human-wt/CIV/6736/2004/G8P[8]
RVA/Human-wt/THA/SKT-457/2014/G8P[8]
RVA/Human-wt/VNM/RVN1293/2014/G8P[8]
RVA/Human-wt/SVN/SI-885/2006/G8P[8]
RVA/Human-wt/HUN/BP1062/2004/G8P[8]
RVA/Human-wt/BRA/5353/2002/G8P[8]
RVA/Human-wt/ARG/Arg16571/2018/G8P[8]
RVA/Human-wt/ARG/Arg15080/2016/G8P[8]
RVA/Human-wt/COD/DRC88/2003/G8P[8]
RVA/Human-wt/JPN/MU14-0/2014/G8P[8]
RVA/Human-tc/IND/69M/1980/G8P[10]
RVA/Human-tc/NGA/HMG035/1999/G8P[1]
RVA/Human-wt/KOR/KJ25-1/xxxx/G8P[7]
RVA/Human-wt/AUS/MG8.01/1996/G8P[14]
RVA/Human-tc/EGY/EGY2295/1998/G8P[14]
RVA/Sheep-tc/ESP/OVR762/2002/G8P[14]
RVA/Cow-tc/JPN/Niigata9801/1987-2000/G8P[11]
RVA/Cow-tc/ZAF/'O'Agent/1965/G8P[1]
RVA/Cow-wt/IND/68/2007/G8P[14]
RVA/Cow-tc/THA/A5-13/1988/G8P[1]
RVA/Cow-tc/NGA/NGRBg8/1998/G8P[1]
RVA/Rhesus-tc/USA/PTRV/1990/G8P[1]
RVA/Guanaco-wt/ARG/Rio_Negro/1998/G8P[1]
RVA/Guanaco-wt/ARG/Chubut/1999/G8P[14]
RVA/Vicuna-wt/ARG/C75/2010/G8P[14]
RVA/Goat-wt/BGD/GBCL0200/G8P[X]
RVA/Fox-wt/HRV/L157-VS/2018/G8P[14]

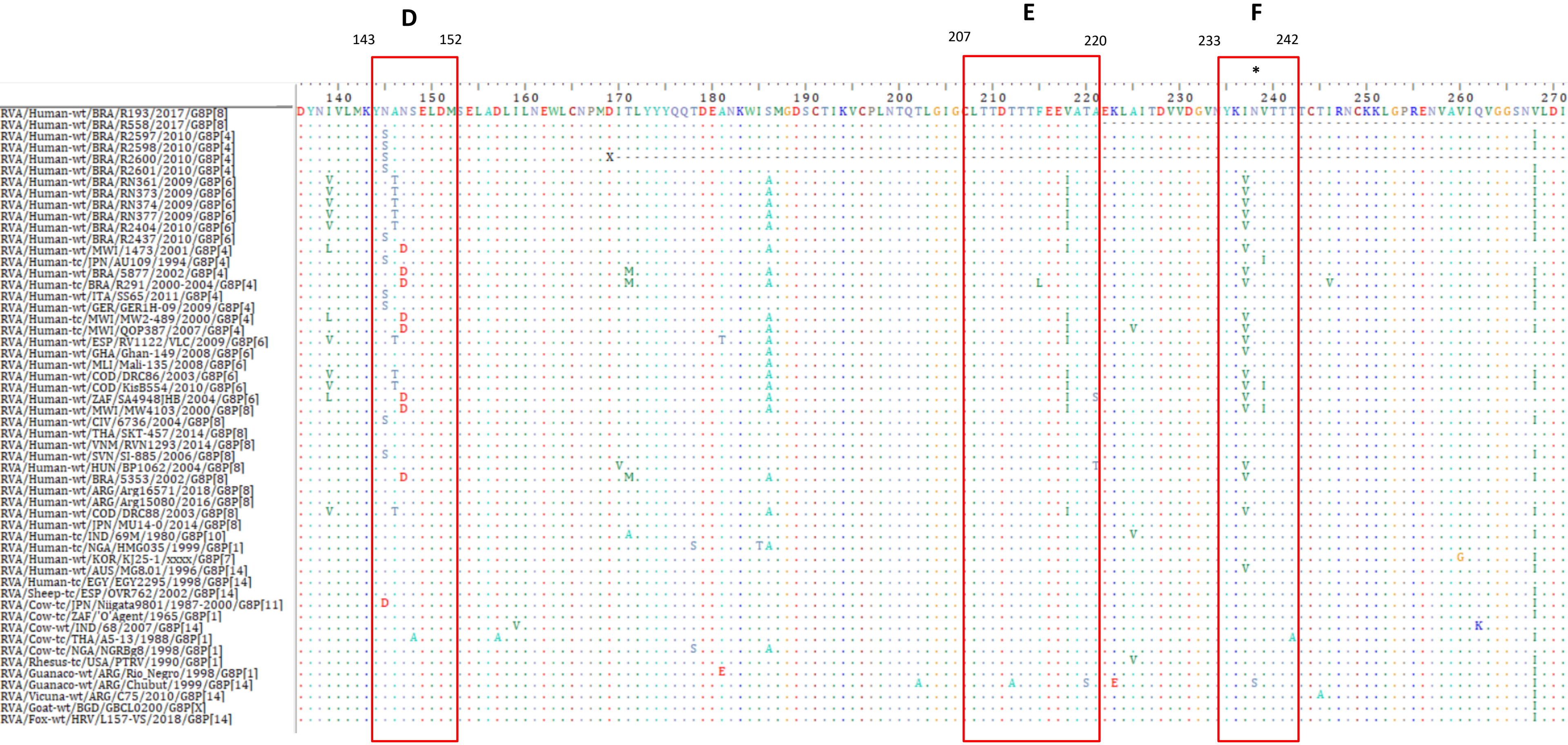
D
E
F
143
152
207
220
233
242
*
RVA/Human-wt/BRA/R193/2017/G8P[8]
RVA/Human-wt/BRA/R558/2017/G8P[8]
RVA/Human-wt/BRA/R2597/2010/G8P[4]
RVA/Human-wt/BRA/R2598/2010/G8P[4]
RVA/Human-wt/BRA/R2600/2010/G8P[4]
RVA/Human-wt/BRA/R2601/2010/G8P[4]
RVA/Human-wt/BRA/RN361/2009/G8P[6]
RVA/Human-wt/BRA/RN373/2009/G8P[6]
RVA/Human-wt/BRA/RN374/2009/G8P[6]
RVA/Human-wt/BRA/RN377/2009/G8P[6]
RVA/Human-wt/BRA/R2404/2010/G8P[6]
RVA/Human-wt/BRA/R2437/2010/G8P[6]
RVA/Human-wt/MWI/1473/2001/G8P[4]
RVA/Human-tc/JPN/AU109/1994/G8P[4]
RVA/Human-wt/BRA/5877/2002/G8P[4]
RVA/Human-tc/BRA/R291/2000-2004/G8P[4]
RVA/Human-wt/ITA/SS65/2011/G8P[4]
RVA/Human-wt/GER/GER1H-09/2009/G8P[4]
RVA/Human-tc/MWI/MW2-489/2000/G8P[4]
RVA/Human-tc/MWI/QOP387/2007/G8P[4]
RVA/Human-wt/ESP/RV1122/VLC/2009/G8P[6]
RVA/Human-wt/GHA/Ghan-149/2008/G8P[6]
RVA/Human-wt/MLI/Mali-135/2008/G8P[6]
RVA/Human-wt/COD/DRC86/2003/G8P[6]
RVA/Human-wt/COD/KisB554/2010/G8P[6]
RVA/Human-wt/ZAF/SA4948JHB/2004/G8P[6]
RVA/Human-wt/MWI/MW4103/2000/G8P[8]
RVA/Human-wt/CIV/6736/2004/G8P[8]
RVA/Human-wt/THA/SKT-457/2014/G8P[8]
RVA/Human-wt/VNM/RVN1293/2014/G8P[8]
RVA/Human-wt/SVN/SI-885/2006/G8P[8]
RVA/Human-wt/HUN/BP1062/2004/G8P[8]
RVA/Human-wt/BRA/5353/2002/G8P[8]
RVA/Human-wt/ARG/Arg16571/2018/G8P[8]
RVA/Human-wt/ARG/Arg15080/2016/G8P[8]
RVA/Human-wt/COD/DRC88/2003/G8P[8]
RVA/Human-wt/JPN/MU14-0/2014/G8P[8]
RVA/Human-tc/IND/69M/1980/G8P[10]
RVA/Human-tc/NGA/HMG035/1999/G8P[1]
RVA/Human-wt/KOR/KJ25-1/xxxx/G8P[7]
RVA/Human-wt/AUS/MG8.01/1996/G8P[14]
RVA/Human-tc/EGY/EGY2295/1998/G8P[14]
RVA/Sheep-tc/ESP/OVR762/2002/G8P[14]
RVA/Cow-tc/JPN/Niigata9801/1987-2000/G8P[11]
RVA/Cow-tc/ZAF/'O'Agent/1965/G8P[1]
RVA/Cow-wt/IND/68/2007/G8P[14]
RVA/Cow-tc/THA/A5-13/1988/G8P[1]
RVA/Cow-tc/NGA/NGRBg8/1998/G8P[1]
RVA/Rhesus-tc/USA/PTRV/1990/G8P[1]
RVA/Guanaco-wt/ARG/Rio_Negro/1998/G8P[1]
RVA/Guanaco-wt/ARG/Chubut/1999/G8P[14]
RVA/Vicuna-wt/ARG/C75/2010/G8P[14]
RVA/Goat-wt/BGD/GBCL0200/G8P[X]
RVA/Fox-wt/HRV/L157-VS/2018/G8P[14]

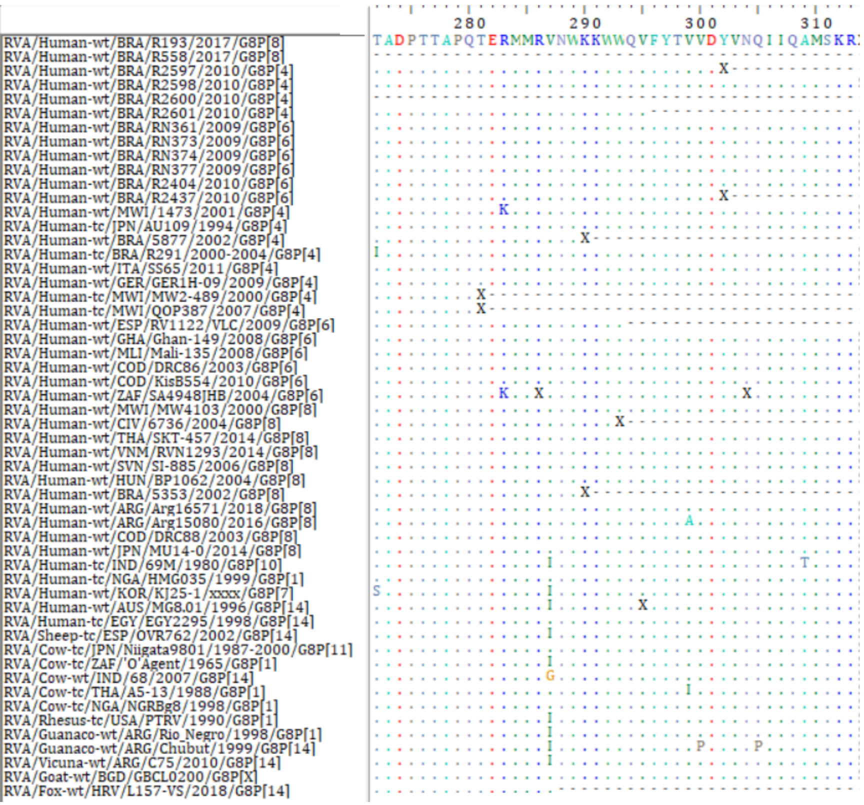

Supplement S2. Deduced amino acid sequence of the VP7 protein of human G8 RVA strains detected in Brazil from 2007-2020. The VP7 antigenic regions A–F are indicated. The N-linked glycosylation sites at positions 69-72 and 238-241 are indicated by asterisks.

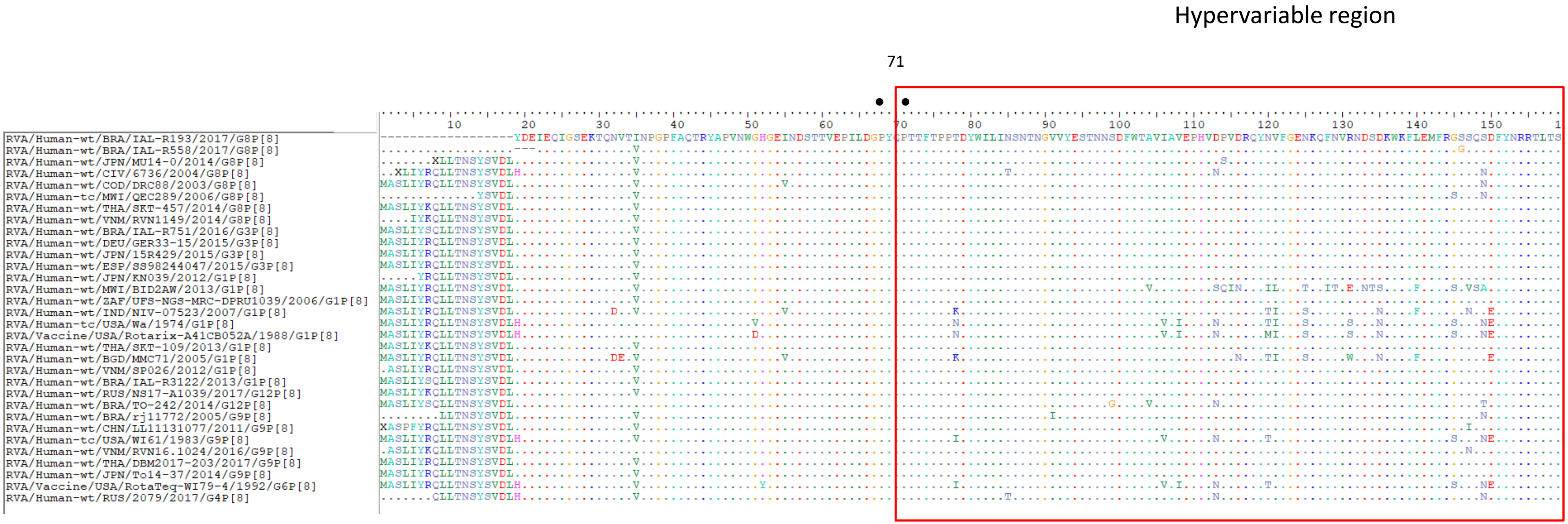
Hypervariable region
71
RVA/Human-wt/BRA/IAL-R193/2017/G8P[8]
RVA/Human-wt/BRA/IAL-R558/2017/G8P[8]
RVA/Human-wt/JPN/MU14-0/2014/G8P[8]
RVA/Human-wt/CIV/6736/2004/G8P[8]
RVA/Human-wt/COD/DRC88/2003/G8P[8]
RVA/Human-tc/MWI/QEC289/2006/G8P[8]
RVA/Human-wt/THA/SKT-457/2014/G8P[8]
RVA/Human-wt/VNM/RVN1149/2014/G8P[8]
RVA/Human-wt/BRA/IAL-R751/2016/G3P[8]
RVA/Human-wt/DEU/GER33-15/2015/G3P[8]
RVA/Human-wt/JPN/15R429/2015/G3P[8]
RVA/Human-wt/ESP/SS98244047/2015/G3P[8]
RVA/Human-wt/JPN/KN039/2012/G1P[8]
RVA/Human-wt/MWI/BID2AW/2013/G1P[8]
RVA/Human-wt/ZAF/UFS-NGS-MRC-DPRU1039/2006/G1P[8]
RVA/Human-wt/IND/NIV-07523/2007/G1P[8]
RVA/Human-tc/USA/Wa/1974/G1P[8]
RVA/Vaccine/USA/Rotarix-A41CB052A/1988/G1P[8]
RVA/Human-wt/THA/SKT-109/2013/G1P[8]
RVA/Human-wt/BGD/MMC71/2005/G1P[8]
RVA/Human-wt/VNM/SP026/2012/G1P[8]
RVA/Human-wt/BRA/IAL-R3122/2013/G1P[8]
RVA/Human-wt/RUS/NS17-A1039/2017/G12P[8]
RVA/Human-wt/BRA/TO-242/2014/G12P[8]
RVA/Human-wt/BRA/rj11772/2005/G9P[8]
RVA/Human-wt/CHN/LL11131077/2011/G9P[8]
RVA/Human-tc/USA/WI61/1983/G9P[8]
RVA/Human-wt/VNM/RVN16.1024/2016/G9P[8]
RVA/Human-wt/THA/DBM2017-203/2017/G9P[8]
RVA/Human-wt/JPN/To14-37/2014/G9P[8]
RVA/Vaccine/USA/RotaTeq-WI79-4/1992/G6P[8]
RVA/Human-wt/RUS/2079/2017/G4P[8]

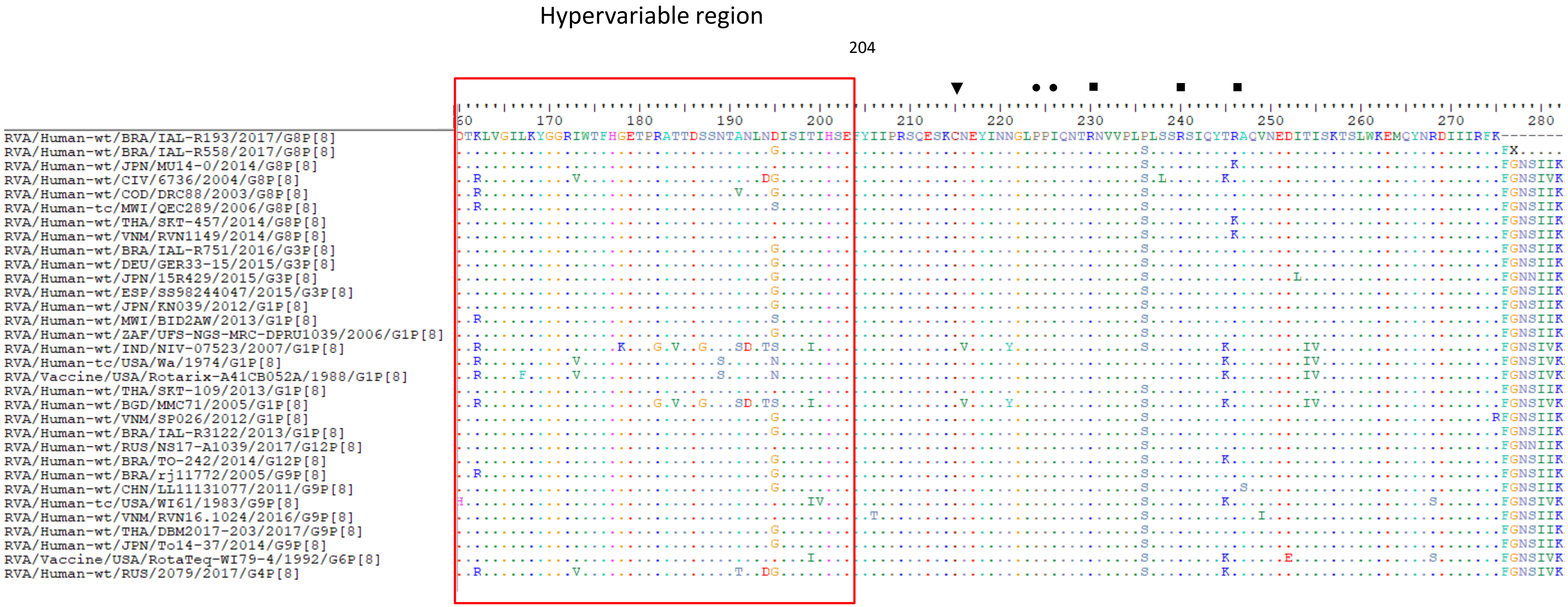

Supplement S3. Deduced amino acid sequence of the VP8* trypsin cleavage product of the VP4 protein of the human rotavirus G8P[8] strains detected in Brazil, 2007-2020, and of a selection of P[8] rotaviruses. The hypervariable region (aa 71–204), and the highly conserved cysteine (▼), proline (●) and arginine (■) are indicated.

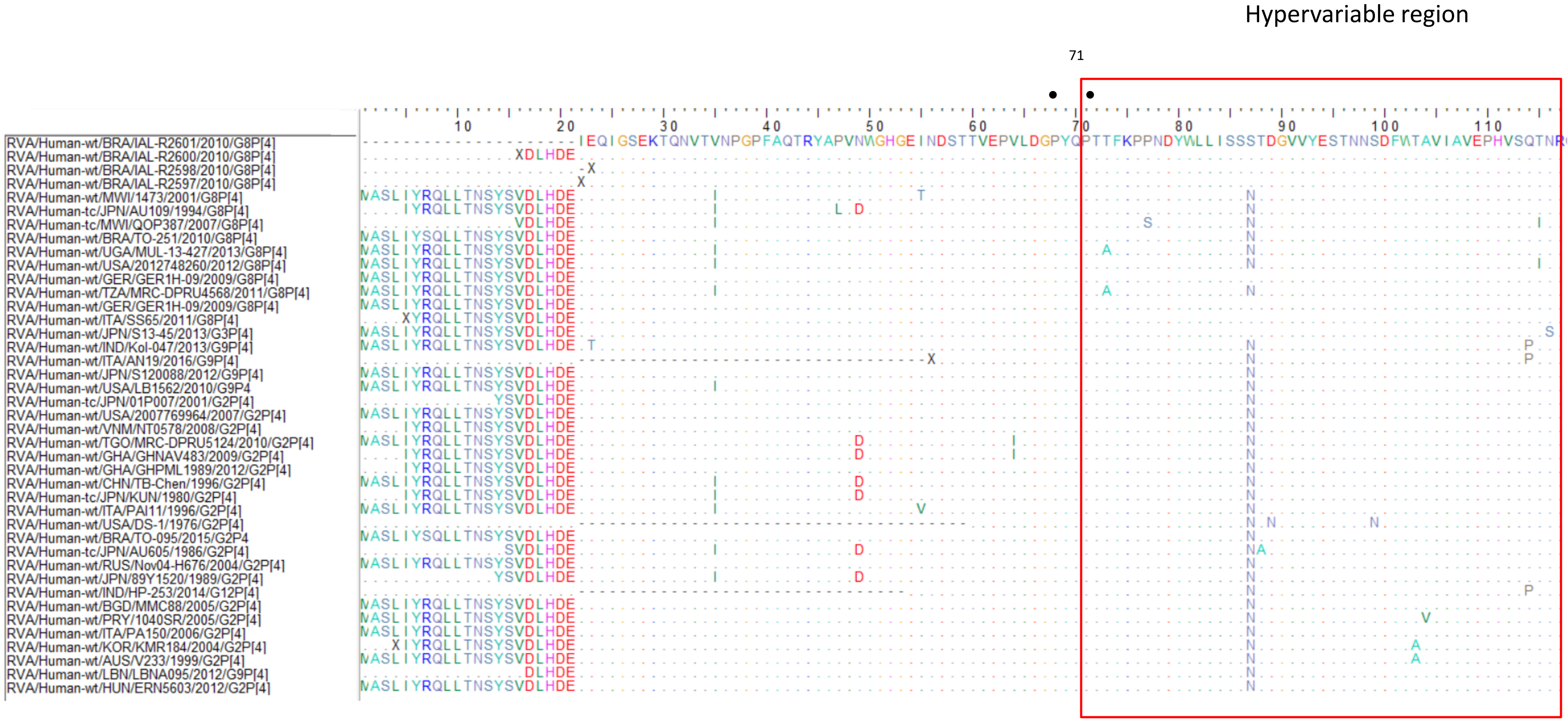

Hypervariable region
71
10 20 30 40 50 60 70 80 90 100 110
IEQIGSEKTQNVTVNPGPFAQTRYAPVNWGHGEINDSTTVEPVLDGPYQPTTFKPPNDYWLLISSSTDGVVYESTNNSDFWTAVIAVEPHVSQTNR
RVA/Human-wt/BRA/IAL-R2601/2010/G8P[4]
RVA/Human-wt/BRA/IAL-R2600/2010/G8P[4]
RVA/Human-wt/BRA/IAL-R2598/2010/G8P[4]
RVA/Human-wt/BRA/IAL-R2597/2010/G8P[4]
RVA/Human-wt/MWI/1473/2001/G8P[4]
RVA/Human-tc/JPN/AU109/1994/G8P[4]
RVA/Human-tc/MWI/QOP387/2007/G8P[4]
RVA/Human-wt/BRA/TO-251/2010/G8P[4]
RVA/Human-wt/UGA/MUL-13-427/2013/G8P[4]
RVA/Human-wt/USA/2012748260/2012/G8P[4]
RVA/Human-wt/GER/GER1H-09/2009/G8P[4]
RVA/Human-wt/TZA/MRC-DPRU4568/2011/G8P[4]
RVA/Human-wt/GER/GER1H-09/2009/G8P[4]
RVA/Human-wt/ITA/SS65/2011/G8P[4]
RVA/Human-wt/JPN/S13-45/2013/G3P[4]
RVA/Human-wt/IND/Kol-047/2013/G9P[4]
RVA/Human-wt/ITA/AN19/2016/G9P[4]
RVA/Human-wt/JPN/S120088/2012/G9P[4]
RVA/Human-wt/USA/LB1562/2010/G9P4
RVA/Human-tc/JPN/01P007/2001/G2P[4]
RVA/Human-wt/USA/2007769964/2007/G2P[4]
RVA/Human-wt/VNM/NT0578/2008/G2P[4]
RVA/Human-wt/TGO/MRC-DPRU5124/2010/G2P[4]
RVA/Human-wt/GHA/GHNAV483/2009/G2P[4]
RVA/Human-wt/GHA/GHPML1989/2012/G2P[4]
RVA/Human-wt/CHN/TB-Chen/1996/G2P[4]
RVA/Human-tc/JPN/KUN/1980/G2P[4]
RVA/Human-wt/ITA/PAI11/1996/G2P[4]
RVA/Human-wt/USA/DS-1/1976/G2P[4]
RVA/Human-wt/BRA/TO-095/2015/G2P4
RVA/Human-tc/JPN/AU605/1986/G2P[4]
RVA/Human-wt/RUS/Nov04-H676/2004/G2P[4]
RVA/Human-wt/JPN/89Y1520/1989/G2P[4]
RVA/Human-wt/IND/HP-253/2014/G12P[4]
RVA/Human-wt/BGD/MMC88/2005/G2P[4]
RVA/Human-wt/PRY/1040SR/2005/G2P[4]
RVA/Human-wt/ITA/PA150/2006/G2P[4]
RVA/Human-wt/KOR/KMR184/2004/G2P[4]
RVA/Human-wt/AUS/V233/1999/G2P[4]
RVA/Human-wt/LBN/LBNA095/2012/G9P[4]
RVA/Human-wt/HUN/ERN5603/2012/G2P[4]

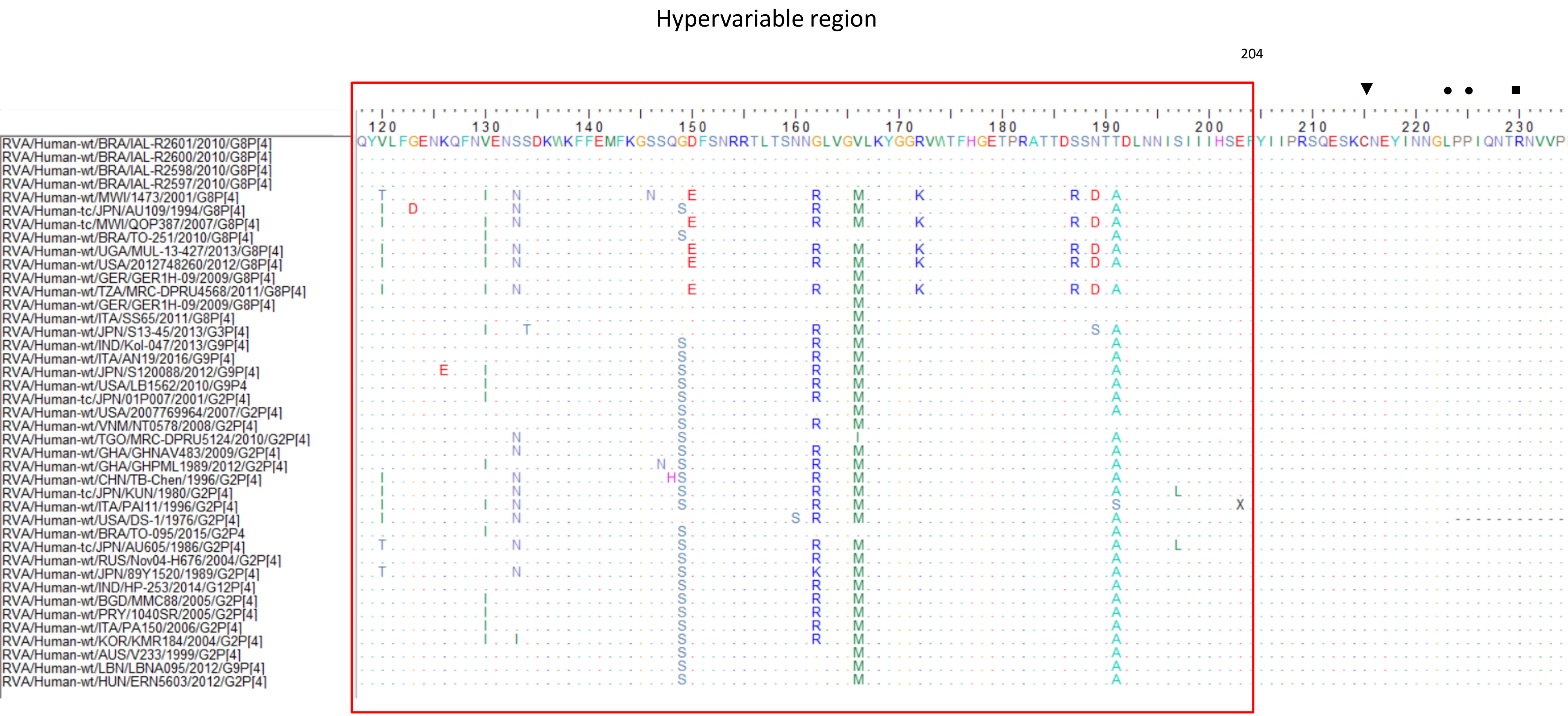

Hypervariable region
204
120 130 140 150 160 170 180 190 200 210 220 230
QYVLFGENKQFNVENSSDKWKFFEMFKGSSQGDFSNRRTLTSNNGLVGVLKYGGRVWTFHGETPRATTDSSNTTDLNNISIIIHSEFYIIPRSQESKCNEYINNGLPPIQNTRNVVP
RVA/Human-wt/BRA/IAL-R2601/2010/G8P[4]
RVA/Human-wt/BRA/IAL-R2600/2010/G8P[4]
RVA/Human-wt/BRA/IAL-R2598/2010/G8P[4]
RVA/Human-wt/BRA/IAL-R2597/2010/G8P[4]
RVA/Human-wt/MWI/1473/2001/G8P[4]
RVA/Human-tc/JPN/AU109/1994/G8P[4]
RVA/Human-tc/MWI/QOP387/2007/G8P[4]
RVA/Human-wt/BRA/TO-251/2010/G8P[4]
RVA/Human-wt/UGA/MUL-13-427/2013/G8P[4]
RVA/Human-wt/USA/2012748260/2012/G8P[4]
RVA/Human-wt/GER/GER1H-09/2009/G8P[4]
RVA/Human-wt/TZA/MRC-DPRU4568/2011/G8P[4]
RVA/Human-wt/GER/GER1H-09/2009/G8P[4]
RVA/Human-wt/ITA/SS65/2011/G8P[4]
RVA/Human-wt/JPN/S13-45/2013/G3P[4]
RVA/Human-wt/IND/Kol-047/2013/G9P[4]
RVA/Human-wt/ITA/AN19/2016/G9P[4]
RVA/Human-wt/JPN/S120088/2012/G9P[4]
RVA/Human-wt/USA/LB1562/2010/G9P4
RVA/Human-tc/JPN/01P007/2001/G2P[4]
RVA/Human-wt/USA/2007769964/2007/G2P[4]
RVA/Human-wt/VNM/NT0578/2008/G2P[4]
RVA/Human-wt/TGO/MRC-DPRU5124/2010/G2P[4]
RVA/Human-wt/GHA/GHNAV483/2009/G2P[4]
RVA/Human-wt/GHA/GHPML1989/2012/G2P[4]
RVA/Human-wt/CHN/TB-Chen/1996/G2P[4]
RVA/Human-tc/JPN/KUN/1980/G2P[4]
RVA/Human-wt/ITA/PAI11/1996/G2P[4]
RVA/Human-wt/USA/DS-1/1976/G2P[4]
RVA/Human-wt/BRA/TO-095/2015/G2P4
RVA/Human-tc/JPN/AU605/1986/G2P[4]
RVA/Human-wt/RUS/Nov04-H676/2004/G2P[4]
RVA/Human-wt/JPN/89Y1520/1989/G2P[4]
RVA/Human-wt/IND/HP-253/2014/G12P[4]
RVA/Human-wt/BGD/MMC88/2005/G2P[4]
RVA/Human-wt/PRY/1040SR/2005/G2P[4]
RVA/Human-wt/ITA/PA150/2006/G2P[4]
RVA/Human-wt/KOR/KMR184/2004/G2P[4]
RVA/Human-wt/AUS/V233/1999/G2P[4]
RVA/Human-wt/LBN/LBNA095/2012/G9P[4]
RVA/Human-wt/HUN/ERN5603/2012/G2P[4]

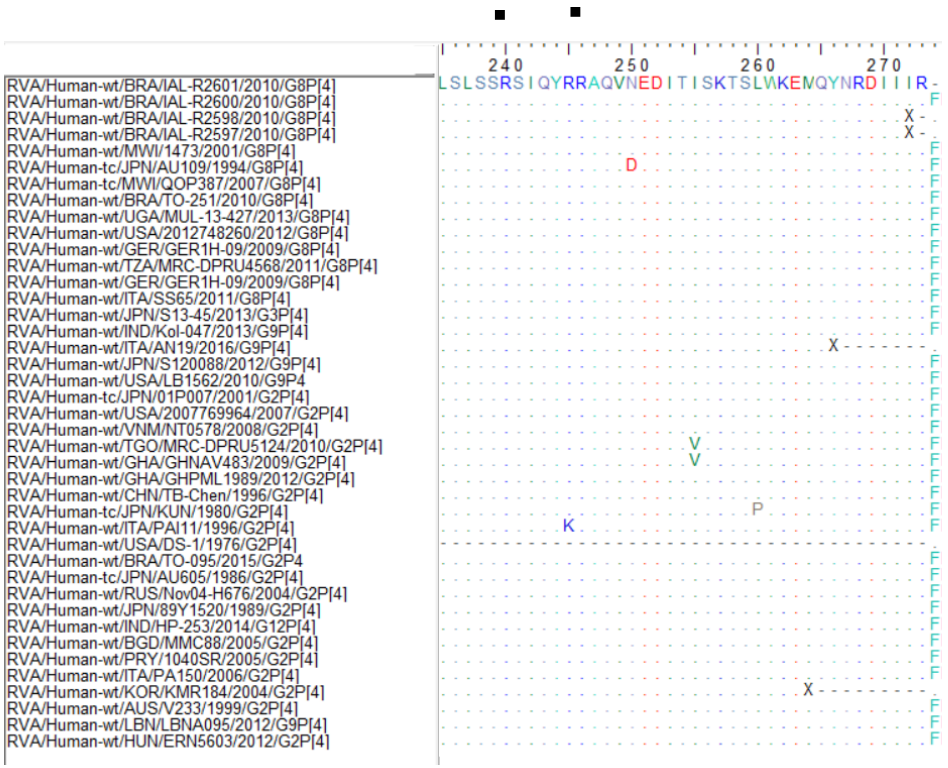

Supplement S4. Deduced amino acid sequence of the VP8* trypsin cleavage product of the VP4 protein of the human rotavirus G8P[4] strains detected in Brazil, 2007-2020, and of a selection of P[4] rotaviruses. The hypervariable region (aa 71–204), and the highly conserved cysteine (▼), proline (●) and arginine (■) are indicated.

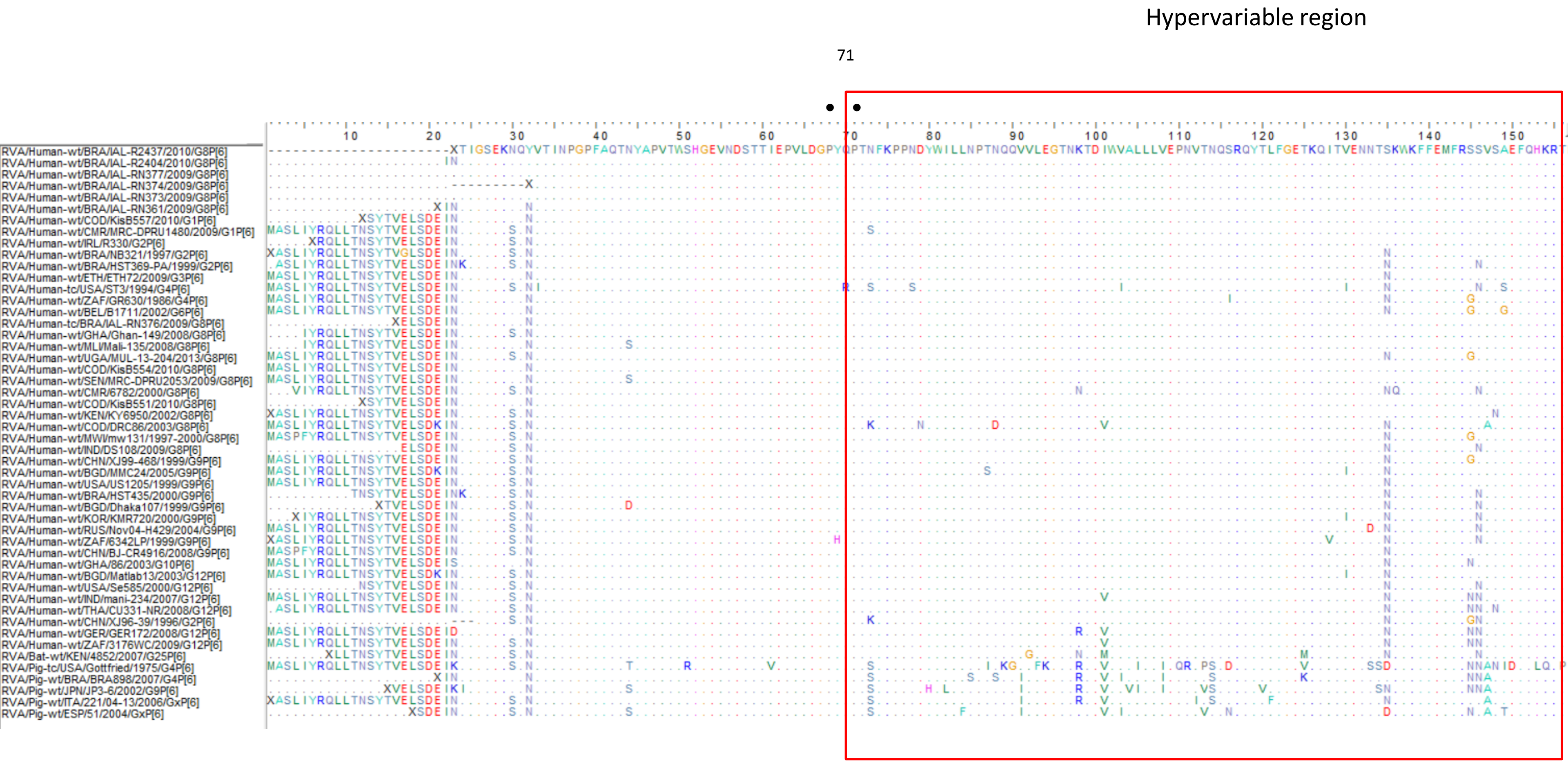

Hypervariable region
71
RVA/Human-wt/BRA/IAL-R2437/2010/G8P[6]
RVA/Human-wt/BRA/IAL-R2404/2010/G8P[6]
RVA/Human-wt/BRA/IAL-RN377/2009/G8P[6]
RVA/Human-wt/BRA/IAL-RN374/2009/G8P[6]
RVA/Human-wt/BRA/IAL-RN373/2009/G8P[6]
RVA/Human-wt/BRA/IAL-RN361/2009/G8P[6]
RVA/Human-wt/COD/KisB557/2010/G1P[6]
RVA/Human-wt/CMR/MRC-DPRU1480/2009/G1P[6]
RVA/Human-wt/IRL/R330/G2P[6]
RVA/Human-wt/BRA/NB321/1997/G2P[6]
RVA/Human-wt/BRA/HST369-PA/1999/G2P[6]
RVA/Human-wt/ETH/ETH72/2009/G3P[6]
RVA/Human-tc/USA/ST3/1994/G4P[6]
RVA/Human-wt/ZAF/GR630/1986/G4P[6]
RVA/Human-wt/BEL/B1711/2002/G6P[6]
RVA/Human-tc/BRA/IAL-RN376/2009/G8P[6]
RVA/Human-wt/GHA/Ghan-149/2008/G8P[6]
RVA/Human-wt/MLI/Mali-135/2008/G8P[6]
RVA/Human-wt/UGA/MUL-13-204/2013/G8P[6]
RVA/Human-wt/COD/KisB554/2010/G8P[6]
RVA/Human-wt/SEN/MRC-DPRU2053/2009/G8P[6]
RVA/Human-wt/CMR/6782/2000/G8P[6]
RVA/Human-wt/COD/KisB551/2010/G8P[6]
RVA/Human-wt/KEN/KY6950/2002/G8P[6]
RVA/Human-wt/COD/DRC86/2003/G8P[6]
RVA/Human-wt/MWI/mw131/1997-2000/G8P[6]
RVA/Human-wt/IND/DS108/2009/G8P[6]
RVA/Human-wt/CHN/XJ99-468/1999/G9P[6]
RVA/Human-wt/BGD/MMC24/2005/G9P[6]
RVA/Human-wt/USA/US1205/1999/G9P[6]
RVA/Human-wt/BRA/HST435/2000/G9P[6]
RVA/Human-wt/BGD/Dhaka107/1999/G9P[6]
RVA/Human-wt/KOR/KMR720/2000/G9P[6]
RVA/Human-wt/RUS/Nov04-H429/2004/G9P[6]
RVA/Human-wt/ZAF/6342LP/1999/G9P[6]
RVA/Human-wt/CHN/BJ-CR4916/2008/G9P[6]
RVA/Human-wt/GHA/86/2003/G10P[6]
RVA/Human-wt/BGD/Matlab13/2003/G12P[6]
RVA/Human-wt/USA/Se585/2000/G12P[6]
RVA/Human-wt/IND/mani-234/2007/G12P[6]
RVA/Human-wt/THA/CU331-NR/2008/G12P[6]
RVA/Human-wt/CHN/XJ96-39/1996/G2P[6]
RVA/Human-wt/GER/GER172/2008/G12P[6]
RVA/Human-wt/ZAF/3176WC/2009/G12P[6]
RVA/Bat-wt/KEN/4852/2007/G25P[6]
RVA/Pig-tc/USA/Gottfried/1975/G4P[6]
RVA/Pig-wt/BRA/BRA898/2007/G4P[6]
RVA/Pig-wt/JPN/JP3-6/2002/G9P[6]
RVA/Pig-wt/ITA/221/04-13/2006/GxP[6]
RVA/Pig-wt/ESP/51/2004/GxP[6]

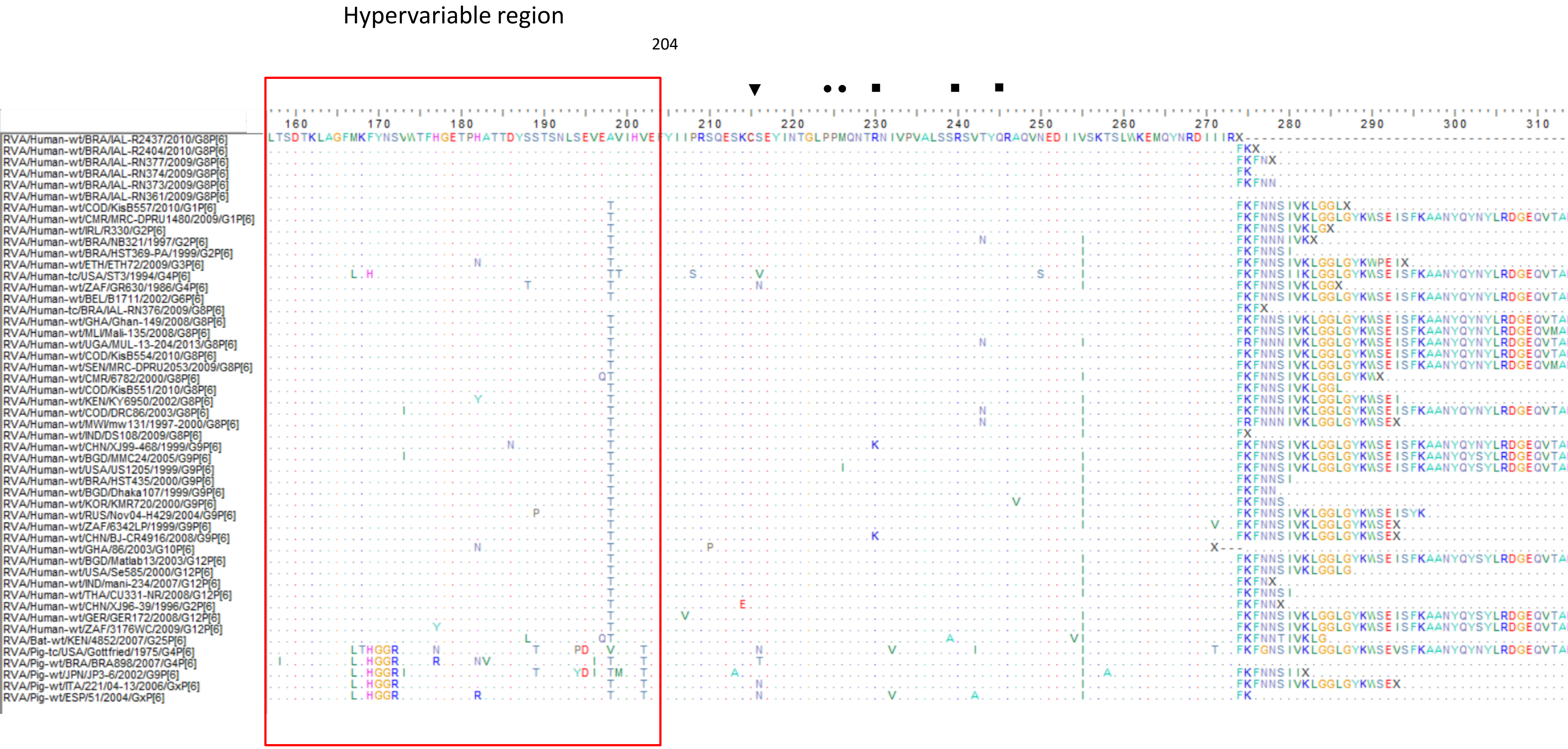

Supplement S5. Deduced amino acid sequence of the VP8* trypsin cleavage product of the VP4 protein of the human rotavirus G8P[6] strains detected in Brazil, 2007-2020, and of a selection of P[6] rotaviruses. The hypervariable region (aa 71–204), and the highly conserved cysteine (▼), proline (●) and arginine (■) are indicated.

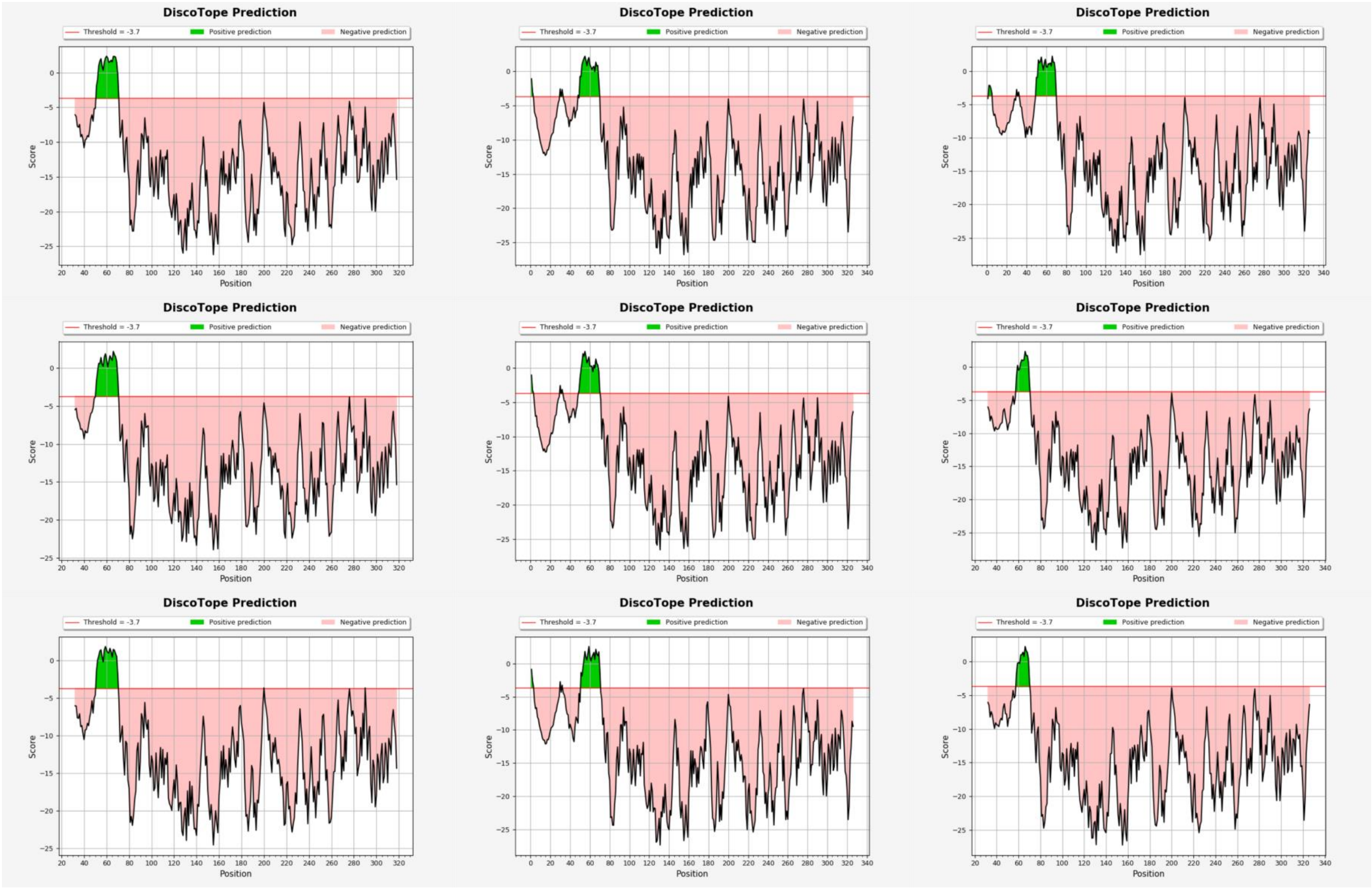

Supplement S6. Conformational B Cell Epitope predictions. Modeled structures were inspected individually for elucidation of potential discontinuous epitopes using IEDB website (https://www.iedb.org/).
